# Supplementary material for: Comparative Mitochondrial Genomes between the Genera Amiota and Phortica (Diptera: Drosophilidae) with Evolutionary Insights into D-Loop Sequence Variability
Source: Genes (Basel). 2023 Jun 9;14(6):1240. doi: 10.3390/genes14061240 (PMC10298597; doi:10.3390/genes14061240)
Supplement: Supplementary file 1 [file genes-14-01240-s001.zip › genes-2404231-supplementary.pdf]

Supplementary Data:

Comparative mitochondrial genomes between the genera *Amiota* and *Phortica* (Diptera: Drosophilidae) with evolutionary insights into D-loop sequence variability

Contents:

Page 2: Table S1. Sizes of the mitogenomes.

Page 3: Table S2. IGNs of the mitogenomes.

Pages 4–5: Table S3. RSCU values of the PCGs in the mitogenomes.

Page 6: Table S4. CDspT values of the PCGs in the mitogenomes.

Pages 7–8: Table S5. Nucleotide compositions and skewness levels of the mitogenomes.

Page 9: Table S6. Ka, Ks, and Ka/Ks values of the PCGs in the mitogenomes.

Page 10: Table S7. Partitioning schemes and data subsets evaluated for the phylogenetic analyses.

Table S1. Sizes of the *Amiota* and *Phortica* mitogenomes.

Gene	*A. dentata*	*A. femorata*	*A. nagatai*	*A. setosa*	*A. spinifemorata*	*A. yifengi*	*P.* (*A.*) *longipenis*	*P.* (*A.*) *tanabei*	*P.* (*P.*) *huazhii*	*P.* (*P.*) *pseudogigas*	*P.* (*P.*) *variegata*	*P.* (*S.*) *hani*
trnI (Ile)	64	65	65	65	65	67	65	66	65	65	65	65
trnQ (Gln)	69	69	69	69	69	69	69	69	69	69	69	69
trnM (Met)	69	69	69	69	69	69	69	69	69	69	69	69
ND2	1,026	1,023	1,026	1,026	1,026	1,023	1,026	1,026	1,026	1,026	1,026	1,026
trnW (Trp)	68	68	68	68	68	68	67	67	68	68	68	67
trnC (Cys)	63	63	64	63	63	62	62	62	63	63	63	62
trnY (Tyr)	65	66	67	66	65	65	68	66	66	65	66	66
COX1	1,539	1,539	1,539	1,539	1,539	1,539	1,539	1,539	1,539	1,539	1,539	1,539
trnL1 (Leu) (UUR)	66	66	66	66	66	66	66	66	66	66	66	66
COX2	681	681	681	681	681	681	685	685	685	685	685	685
trnK (Lys)	71	71	71	71	71	71	71	71	71	71	71	71
trnD (Asp)	70	70	67	70	66	70	67	67	66	67	67	66
ATP8	162	162	162	162	162	162	162	162	162	162	162	162
ATP6	678	678	678	678	678	678	678	678	678	678	678	678
COX3	789	789	789	789	789	789	789	789	789	789	789	789
trnG (Gly)	64	64	66	65	65	64	64	65	65	64	65	64
ND3	354	354	354	352	354	354	354	354	354	354	354	354
trnA (Ala)	65	67	65	66	65	65	66	65	65	65	65	65
trnR (Arg)	64	64	63	63	63	63	64	63	63	63	63	63
trnN (Asn)	69	65	65	66	65	65	66	67	65	65	65	65
trnS2 (Ser) (AGN)	66	68	67	66	68	68	67	67	67	65	67	67
trnE (Glu)	67	68	67	66	67	67	67	67	66	66	66	66
trnF (Phe)	66	66	66	68	66	66	66	66	66	67	67	66
ND5	1,717	1,720	1,720	1,717	1,720	1,720	1,722	1,722	1,720	1,720	1,720	1,720
trnH (His)	65	65	66	66	66	66	66	65	66	65	65	65
ND4	1,339	1,339	1,339	1,339	1,339	1,339	1,339	1,339	1,339	1,339	1,339	1,341
ND4L	291	291	291	291	291	291	291	291	291	291	291	291
trnT (Thr)	64	65	64	65	65	65	65	65	65	65	65	65
trnP (Pro)	67	66	67	66	66	66	66	66	68	67	66	66
ND6	525	525	525	525	525	525	525	525	525	525	525	525
CYTB	1,137	1,137	1,137	1,137	1,137	1,137	1,137	1,137	1,137	1,137	1,137	1,137
trnS1 (Ser) (UCN)	67	67	67	68	67	67	66	67	67	67	67	67
ND1	937	937	937	916	937	937	937	937	937	937	937	937
trnL2 (Leu) (CUN)	65	66	65	67	65	67	63	64	63	64	64	64
lrRNA	1,329	1,327	1,330	1,322	1,338	1,328	1,323	1,318	1,320	1,322	1,320	1,317
trnV (Val)	72	72	72	72	72	72	72	72	72	72	72	72
srRNA	787	784	789	785	787	783	785	784	788	787	788	787
D-loop region	1,912	1,672	1,554	1,995	1,319	1,515	1,355	1,084	1,182	1,158	1,134	1,118

Table S2. Intergenic nucleotides (IGNs) of the *Amiota* and *Phortica* mitogenomes.

Gene	*A. dentata*	*A. femorata*	*A. nagatai*	*A. setosa*	*A. spinifemorata*	*A. yifengi*	*P.* (*A.*) *longipenis*	*P.* (*A.*) *tanabei*	*P.* (*P.*) *huazhii*	*P.* (*P.*) *pseudogigas*	*P.* (*P.*) *variegata*	*P.* (*S.*) *hani*
trnI (Ile)	0	7	0	1	1	0	100	14	15	15	15	13
trnQ (Gln)	−1	−1	5	−1	−1	−1	−1	−1	−1	−1	−1	−1
trnM (Met)	0	0	0	0	0	0	0	0	0	0	0	0
ND2	0	0	0	0	0	0	0	0	0	0	0	0
trnW (Trp)	6	6	38	6	6	6	6	6	6	6	6	6
trnC (Cys)	2	2	47	0	2	2	1	4	2	2	2	2
trnY (Tyr)	0	0	0	0	0	0	0	0	0	0	0	0
COX1	3	3	3	3	3	3	3	3	3	3	3	3
trnL1 (Leu) (UUR)	2	3	6	3	4	3	2	5	2	6	2	2
COX2	4	7	5	6	6	6	3	3	3	3	3	3
trnK (Lys)	1	−1	0	−1	0	2	3	0	2	−1	0	0
trnD (Asp)	0	0	0	0	1	0	0	0	0	0	0	0
ATP8	5	5	5	5	5	5	5	5	5	5	5	5
ATP6	−1	−1	−1	−1	−1	−1	−1	5	9	12	6	11
COX3	8	8	11	8	8	8	9	9	9	9	9	9
trnG (Gly)	0	0	0	0	0	0	0	0	0	0	0	0
ND3	0	0	3	0	0	0	1	2	0	0	0	0
trnA (Ala)	2	8	49	2	2	3	−1	−1	−1	−1	−1	−1
trnR (Arg)	0	3	32	2	1	1	7	9	7	2	2	3
trnN (Asn)	1	0	0	1	0	0	0	0	0	1	0	0
trnS2 (Ser) (AGN)	1	0	1	1	0	0	0	0	0	1	0	0
trnE (Glu)	18	18	18	18	18	18	18	18	19	18	18	18
trnF (Phe)	0	0	0	0	0	0	19	9	0	0	0	0
ND5	15	15	15	15	15	15	15	15	15	15	15	15
trnH (His)	0	0	0	0	0	0	0	0	0	0	0	−1
ND4	4	2	6	1	3	4	−1	−1	−1	−1	0	−1
ND4L	2	2	2	2	2	2	2	2	2	2	2	2
trnT (Thr)	0	0	0	0	0	0	0	0	0	0	0	0
trnP (Pro)	2	3	3	2	2	2	2	2	2	2	2	2
ND6	−1	−1	6	−1	−1	−1	−1	−1	−1	−1	−1	−1
CYTB	0	0	−1	0	0	0	0	4	0	0	0	6
trnS1 (Ser) (UCN)	15	15	15	15	15	15	18	18	18	18	18	10
ND1	10	10	10	31	10	10	10	10	10	10	10	10
trnL2 (Leu) (CUN)	0	0	0	0	0	0	0	0	0	0	0	0
lrRNA	0	0	0	0	0	0	0	0	0	0	0	0
trnV (Val)	0	0	0	0	0	0	0	0	0	0	0	0
srRNA	0	0	0	0	0	0	0	0	0	0	0	0
D-loop region	0	0	0	0	0	0	0	0	0	0	0	0

Table S3. Relative synonymous codon usage (RSCU) values of the protein-coding genes (PCGs) in the *Amiota* and *Phortica* mitogenomes.

Amino acid	Codon	*A. dentata*	*A. femorata*	*A. nagatai*	*A. setosa*	*A. spinifemorata*	*A. yifengi*	*P.* (*A.*) *longipenis*	*P.* (*A.*) *tanabei*	*P.* (*P.*) *huazhii*	*P.* (*P.*) *pseudogigas*	*P.* (*P.*) *variegata*	*P.* (*S.*) *hani*
Ala	GCA	1.13	1.53	1.61	1.46	1.19	1.27	1.48	1.30	1.25	1.30	1.06	1.16
	GCU	2.44	2.40	2.34	2.17	2.53	2.47	2.29	2.47	2.45	2.38	2.58	2.61
	GCG	0.05	0.05	0.00	0.10	0.10	0.03	0.02	0.05	0.07	0.07	0.04	0.05
	GCC	0.38	0.02	0.05	0.27	0.17	0.23	0.21	0.18	0.23	0.25	0.31	0.18
Arg	CGA	2.51	2.85	2.58	2.69	2.85	2.71	2.78	2.98	2.71	2.71	2.78	2.78
	CGU	1.22	0.81	1.36	1.24	0.95	0.95	1.08	0.95	0.95	1.15	1.02	1.08
	CGG	0.14	0.27	0.07	0.07	0.20	0.14	0.14	0.07	0.34	0.14	0.20	0.14
	CGC	0.14	0.07	0.00	0.00	0.00	0.20	0.00	0.00	0.00	0.00	0.00	0.00
Asn	AAU	1.97	1.90	1.88	1.90	1.88	1.89	1.84	1.94	1.71	1.82	1.81	1.85
	AAC	0.10	0.10	0.13	0.10	0.12	0.11	0.16	0.06	0.29	0.18	0.19	0.15
Asp	GAU	1.77	1.84	1.78	1.85	1.81	1.81	1.70	1.82	1.42	1.55	1.55	1.76
	GAC	0.23	0.16	0.22	0.15	0.19	0.19	0.30	0.18	0.58	0.45	0.45	0.24
Cys	UGU	1.95	2.00	2.00	1.95	1.94	1.95	1.90	1.90	1.95	2.00	1.90	1.74
	UGC	0.05	0.00	0.00	0.05	0.06	0.05	0.10	0.10	0.05	0.00	0.10	0.26
Gln	CAA	1.83	1.97	1.94	1.81	1.94	1.88	1.97	1.89	1.86	1.86	1.94	1.86
	CAG	0.17	0.03	0.06	0.19	0.06	0.12	0.03	0.11	0.14	0.14	0.06	0.14
Glu	GAA	1.88	1.85	1.90	1.84	1.90	1.95	1.87	1.97	1.84	1.82	1.89	1.95
	GAG	0.12	0.15	0.10	0.16	0.10	0.05	0.13	0.03	0.16	0.18	0.11	0.05
Gly	GGA	2.66	2.67	2.64	2.42	2.52	2.70	2.72	2.76	2.70	2.73	2.61	2.67
	GGU	0.83	1.00	1.10	1.19	1.10	0.97	1.00	1.04	0.68	0.94	0.97	1.03
	GGG	0.44	0.29	0.20	0.29	0.29	0.22	0.24	0.19	0.57	0.29	0.38	0.28
	GGC	0.07	0.04	0.05	0.09	0.09	0.11	0.04	0.02	0.06	0.04	0.04	0.02
His	CAU	1.48	1.71	1.82	1.50	1.76	1.70	1.61	1.79	1.37	1.45	1.47	1.63
	CAC	0.52	0.29	0.18	0.50	0.24	0.30	0.39	0.21	0.63	0.55	0.53	0.37
Ile	AUU	1.95	1.95	1.97	1.90	1.92	1.91	1.96	1.94	1.84	1.85	1.89	1.95
	AUC	0.05	0.05	0.03	0.10	0.08	0.09	0.04	0.06	0.16	0.15	0.11	0.05
Leu1	UUA	5.02	5.21	5.32	4.94	5.19	5.33	5.08	5.20	4.75	4.77	4.83	5.09
	UUG	0.40	0.25	0.15	0.31	0.27	0.23	0.27	0.29	0.36	0.40	0.26	0.19
Leu2	CUA	0.27	0.22	0.19	0.45	0.22	0.20	0.22	0.16	0.39	0.26	0.40	0.25
	CUU	0.29	0.30	0.33	0.25	0.28	0.22	0.42	0.32	0.48	0.53	0.47	0.43
	CUG	0.00	0.01	0.01	0.01	0.01	0.02	0.00	0.03	0.02	0.01	0.02	0.02
	CUC	0.02	0.00	0.00	0.04	0.03	0.01	0.00	0.00	0.00	0.03	0.02	0.02
Lys	AAA	1.88	1.86	1.78	1.75	1.89	1.83	1.89	1.87	1.61	1.60	1.73	1.57
	AAG	0.12	0.14	0.22	0.25	0.11	0.17	0.11	0.13	0.39	0.40	0.27	0.43
Met	AUA	1.79	1.89	1.84	1.84	1.81	1.85	1.85	1.84	1.84	1.76	1.77	1.81
	AUG	0.21	0.11	0.16	0.16	0.19	0.15	0.15	0.16	0.16	0.24	0.23	0.19
Phe	UUU	1.92	1.89	1.94	1.90	1.95	1.92	1.88	1.91	1.82	1.88	1.82	1.83
	UUC	0.08	0.11	0.06	0.10	0.05	0.08	0.12	0.09	0.18	0.12	0.18	0.17
Pro	CCA	1.15	1.13	1.48	1.10	0.95	1.27	0.71	0.86	1.13	0.89	1.04	0.85
	CCU	2.51	2.69	2.49	2.41	2.74	2.45	3.01	2.80	2.50	2.75	2.66	2.78
	CCG	0.09	0.03	0.00	0.00	0.12	0.00	0.00	0.09	0.15	0.12	0.06	0.09
	CCC	0.25	0.15	0.03	0.49	0.18	0.28	0.28	0.25	0.21	0.24	0.24	0.27

Ser1	UCA	2.57	2.12	2.35	2.04	2.37	2.37	2.00	2.12	2.20	2.48	2.28	2.26
	UCU	2.83	3.30	3.10	3.20	3.00	2.99	3.20	3.13	2.85	2.79	2.96	3.04
	UCG	0.09	0.02	0.05	0.09	0.07	0.12	0.07	0.07	0.23	0.14	0.07	0.12
	UCC	0.09	0.09	0.05	0.26	0.16	0.22	0.05	0.02	0.12	0.07	0.09	0.02
Ser2	AGA	1.64	1.53	1.65	1.56	1.58	1.60	1.51	1.48	1.45	1.48	1.53	1.41
	AGU	0.63	0.88	0.75	0.76	0.77	0.65	1.13	1.08	0.98	0.93	1.04	1.15
	AGG	0.00	0.00	0.00	0.00	0.00	0.00	0.00	0.00	0.00	0.00	0.00	0.00
	AGC	0.14	0.05	0.05	0.09	0.05	0.05	0.05	0.09	0.16	0.12	0.02	0.00
Thr	ACA	1.95	1.97	2.02	1.58	1.96	1.72	1.72	1.58	1.74	1.70	1.76	1.86
	ACU	1.77	1.94	1.93	2.09	1.94	2.15	2.17	2.20	2.07	2.06	2.04	1.97
	ACG	0.02	0.04	0.02	0.09	0.00	0.09	0.04	0.07	0.04	0.15	0.07	0.02
	ACC	0.26	0.04	0.02	0.24	0.09	0.04	0.07	0.15	0.17	0.09	0.13	0.15
Trp	UGA	1.92	1.96	1.96	1.88	1.86	1.92	1.94	1.96	1.96	1.96	1.98	1.94
	UGG	0.08	0.04	0.04	0.12	0.14	0.08	0.06	0.04	0.04	0.04	0.02	0.06
Tyr	UAU	1.85	1.89	1.95	1.87	1.83	1.81	1.87	1.84	1.64	1.66	1.63	1.72
	UAC	0.15	0.11	0.05	0.13	0.17	0.19	0.13	0.16	0.36	0.34	0.37	0.28
Val	GUA	1.57	1.71	1.76	2.01	1.79	1.47	1.66	1.73	1.94	1.99	1.74	2.01
	GUU	2.24	2.13	2.17	1.82	2.07	2.31	2.10	2.01	1.72	1.88	2.11	1.87
	GUG	0.17	0.11	0.02	0.09	0.07	0.14	0.17	0.20	0.20	0.09	0.12	0.12
	GUC	0.02	0.05	0.04	0.07	0.07	0.07	0.06	0.07	0.14	0.04	0.04	0.00

Table S4. Codons per thousand codon (CDspT) values of the PCGs in the *Amiota* and *Phortica* mitogenomes.

Amino acid	*A. dentata*	*A. femorata*	*A. nagatai*	*A. setosa*	*A. spinifemorata*	*A. yifengi*	*P.* (*A.*) *longipenis*	*P.* (*A.*) *tanabei*	*P.* (*P.*) *huazhii*	*P.* (*P.*) *pseudogigas*	*P.* (*P.*) *variegata*	*P.* (*S.*) *hani*
Ala	42.85	43.65	43.37	43.74	43.37	41.50	45.23	47.12	46.58	47.12	47.93	46.58
Arg	15.90	15.90	15.89	15.66	15.89	15.90	15.89	15.89	15.89	15.89	15.89	15.89
Asn	56.59	56.05	56.03	56.70	58.73	56.32	54.93	53.85	53.58	53.85	53.58	53.85
Asp	16.44	17.25	16.97	18.09	16.97	16.98	17.77	17.50	17.77	17.77	17.77	17.77
Cys	10.78	10.24	10.51	10.53	9.16	10.78	10.77	10.77	11.04	11.04	11.31	10.50
Glu	22.64	21.83	22.36	20.52	22.09	22.64	20.46	20.73	20.19	20.46	20.19	20.19
Gly	58.74	59.01	59.54	58.86	59.00	57.94	58.16	58.16	58.70	58.43	58.97	58.43
His	19.67	20.75	20.47	20.52	19.94	19.94	20.46	20.46	20.46	20.46	20.46	20.46
Ile	103.48	100.51	101.56	102.32	101.56	106.17	98.55	99.89	95.58	93.97	94.24	96.93
Leu1	148.48	150.63	150.32	144.71	150.05	151.71	147.82	152.40	142.16	143.24	142.16	147.82
Leu2	15.63	14.82	14.55	20.52	14.82	12.13	17.77	14.27	24.77	22.89	25.31	20.19
Lys	23.17	23.17	24.78	23.76	23.98	25.33	24.77	24.23	23.69	24.23	23.96	23.96
Met	59.28	61.71	59.27	60.48	60.88	61.17	62.47	58.97	56.27	56.54	55.74	56.54
Phe	94.04	89.73	92.13	91.52	90.79	93.24	88.58	89.39	89.12	89.12	89.12	89.93
Pro	34.76	35.30	35.02	35.37	35.02	34.76	34.73	35.00	35.27	35.27	35.27	35.27
Ser1	64.40	64.13	61.69	63.71	64.92	64.13	60.85	61.12	62.20	61.93	61.93	62.20
Ser2	27.76	28.56	27.21	27.54	27.75	25.87	30.69	30.43	29.89	28.54	29.62	29.35
Thr	46.35	48.23	46.88	45.90	46.07	48.23	48.20	49.00	50.35	50.08	49.54	49.27
Trp	26.95	26.41	26.40	27.00	26.94	26.68	26.66	26.66	26.93	26.66	26.93	26.66
Tyr	47.97	45.54	46.88	46.98	46.61	45.27	46.58	45.77	46.31	45.77	45.50	46.85
Val	45.27	47.16	49.57	46.17	46.34	44.73	49.81	49.27	53.85	57.35	55.20	51.97

Table S5. Nucleotide compositions and skewness levels of the *Amiota* and *Phortica* mitogenomes.

Regions/genes	Species	Size (bp)	Nucleotides composition (%)				AT-skew	GC-skew
			A	T	G	C		
Whole mitogenome (J-strands)	*A. dentata*	16,725	40.7	39.2	8.1	12.0	0.019	−0.194
	A. femorata	16,499	40.0	39.9	8.6	11.4	0.001	−0.140
	A. nagatai	16,571	40.2	40.3	8.4	11.2	−0.001	−0.143
	A. setosa	16,803	40.7	38.8	8.1	12.4	0.024	−0.210
	A. spinifemorata	16,143	40.2	39.4	8.7	11.7	0.010	−0.147
	A. yifengi	16,329	40.4	39.7	8.3	11.7	0.009	−0.170
	P. (*A.*) *longipenis*	16,290	40.1	39.4	8.4	12.1	0.009	−0.180
	P. (*A.*) *tanabei*	15,933	39.9	39.2	8.8	12.0	0.009	−0.154
	P. (*P.*) *huazhii*	16,016	39.1	38.1	9.1	13.6	0.013	−0.198
	P. (*P.*) *pseudogigas*	15,990	39.1	38.4	9.5	12.9	0.009	−0.152
	P. (*P.*) *variegata*	15,958	39.3	38.5	9.2	13.0	0.010	−0.171
	P. (*S.*) *hani*	15,937	39.4	39.0	9.1	12.5	0.005	−0.157
PCGs	*A. dentata*	11,175	32.3	44.9	12.0	10.9	−0.163	0.048
	A. femorata	11,175	32.8	44.9	11.7	10.6	−0.156	0.049
	A. nagatai	11,178	32.9	45.2	11.6	10.2	−0.157	0.064
	A. setosa	11,152	32.2	44.6	11.9	11.3	−0.161	0.026
	A. spinifemorata	11,178	32.6	44.9	11.8	10.7	−0.159	0.049
	A. yifengi	11,175	32.7	45.0	11.5	10.7	−0.158	0.036
	P. (*A.*) *longipenis*	11,184	32.4	44.9	11.9	10.8	−0.162	0.048
	P. (*A.*) *tanabei*	11,184	32.3	45.1	12.0	10.7	−0.165	0.057
	P. (*P.*) *huazhii*	11,182	31.8	43.4	12.7	12.2	−0.154	0.020
	P. (*P.*) *pseudogigas*	11,182	31.7	43.9	12.6	11.7	−0.161	0.037
	P. (*P.*) *variegata*	11,182	31.7	44.0	12.4	11.9	−0.162	0.021
	P. (*S.*) *hani*	11,184	32.0	44.6	12.1	11.3	−0.164	0.034
PCGs (1st codon positions)	*A. dentata*	3,727	31.6	39.5	18.5	10.4	−0.111	0.280
	A. femorata	3,727	31.7	38.9	18.8	10.6	−0.102	0.279
	A. nagatai	3,728	31.5	39.0	19.1	10.4	−0.106	0.295
	A. setosa	3,720	31.6	38.7	18.7	11.1	−0.101	0.255
	A. spinifemorata	3,728	31.8	39.1	18.7	10.4	−0.103	0.285
	A. yifengi	3,727	32.2	39.4	18.3	10.1	−0.101	0.289
	P. (*A.*) *longipenis*	3,730	31.8	38.3	19.1	10.7	−0.093	0.282
	P. (*A.*) *tanabei*	3,730	31.5	38.8	19.2	10.4	−0.104	0.297
	P. (*P.*) *huazhii*	3,730	30.8	38.0	19.7	11.5	−0.105	0.263
	P. (*P.*) *pseudogigas*	3,730	30.6	38.0	20.1	11.3	−0.108	0.280
	P. (*P.*) *variegata*	3,730	30.6	37.9	19.9	11.6	−0.107	0.263
	P. (*S.*) *hani*	3,730	30.9	38.6	19.4	11.1	−0.111	0.272
PCGs (2nd codon positions)	*A. dentata*	3,724	20.8	46.5	14.0	18.8	−0.382	−0.146
	A. femorata	3,724	20.6	46.3	14.0	19.1	−0.384	−0.154
	A. nagatai	3,725	20.9	46.6	13.9	18.6	−0.381	−0.145
	A. setosa	3,716	20.8	46.4	13.9	18.8	−0.381	−0.150
	A. spinifemorata	3,725	21.0	46.3	13.8	18.9	−0.376	−0.156
	A. yifengi	3,724	20.8	46.8	13.7	18.8	−0.385	−0.157
	P. (*A.*) *longipenis*	3,727	20.6	46.4	14.2	18.8	−0.385	−0.139
	P. (*A.*) *tanabei*	3,727	20.4	46.3	14.1	19.2	−0.388	−0.153
	P. (*P.*) *huazhii*	3,726	20.4	46.1	14.2	19.4	−0.386	−0.155
	P. (*P.*) *pseudogigas*	3,726	20.4	46.2	14.0	19.4	−0.387	−0.162
	P. (*P.*) *variegata*	3,726	20.3	46.1	14.2	19.4	−0.389	−0.155
	P. (*S.*) *hani*	3,727	20.5	46.2	14.0	19.3	−0.385	−0.159
PCGs (3rd codon positions)	*A. dentata*	3,724	44.5	48.6	3.5	3.4	−0.044	0.014
	A. femorata	3,724	45.9	49.5	2.4	2.2	−0.038	0.043
	A. nagatai	3,725	46.4	50.1	1.8	1.6	−0.038	0.059
	A. setosa	3,716	44.2	48.7	3.1	4.0	−0.048	−0.127
	A. spinifemorata	3,725	45.0	49.4	2.8	2.8	−0.047	0.000
	A. yifengi	3,724	45.3	49.0	2.5	3.2	−0.039	−0.123
	P. (*A.*) *longipenis*	3,727	44.8	49.9	2.4	3.0	−0.054	−0.111
	P. (*A.*) *tanabei*	3,727	44.9	50.1	2.5	2.5	−0.055	0.000
	P. (*P.*) *huazhii*	3,726	44.2	46.1	4.1	5.6	−0.021	−0.155
	P. (*P.*) *pseudogigas*	3,726	44.0	47.6	3.9	4.5	−0.039	−0.071
	P. (*P.*) *variegata*	3,726	44.2	48.0	3.1	4.7	−0.041	−0.205
	P. (*S.*) *hani*	3,727	44.6	49.0	2.9	3.5	−0.047	−0.094

**tRNAs**	*A. dentata*	1,444	38.7	39.4	12.5	9.3	−0.009	0.147
	A. femorata	1,448	39.0	39.0	12.6	9.4	0.000	0.145
	A. nagatai	1,444	38.4	39.8	12.7	9.1	−0.018	0.165
	A. setosa	1,449	38.7	39.9	12.2	9.2	−0.015	0.140
	A. spinifemorata	1,440	38.0	39.7	13.1	9.2	−0.022	0.175
	A. yifengi	1,446	38.9	39.4	12.6	9.1	−0.006	0.161
	P. (*A.*) *longipenis*	1,440	38.8	39.3	12.2	9.7	−0.006	0.114
	P. (*A.*) *tanabei*	1,440	38.2	39.2	12.7	9.9	−0.013	0.124
	P. (*P.*) *huazhii*	1,439	38.2	38.2	13.4	10.2	0.000	0.136
	P. (*P.*) *pseudogigas*	1,436	38.0	39.0	13.2	9.8	−0.013	0.148
	P. (*P.*) *variegata*	1,439	37.7	38.6	13.5	10.2	−0.012	0.139
	P. (*S.*) *hani*	1,434	38.4	38.9	12.8	9.8	−0.006	0.133
**rRNAs**	*A. dentata*	2,116	41.3	41.0	11.7	6.0	0.004	0.322
	A. femorata	2,111	41.2	40.8	11.6	6.4	0.005	0.289
	A. nagatai	2,119	41.0	41.2	11.8	6.1	−0.002	0.318
	A. setosa	2,107	40.4	41.0	12.3	6.3	−0.007	0.323
	A. spinifemorata	2,125	41.4	40.9	11.5	6.2	0.006	0.299
	A. yifengi	2,111	41.4	41.1	11.5	6.1	0.004	0.307
	P. (*A.*) *longipenis*	2,108	40.3	42.3	11.3	6.1	−0.024	0.299
	P. (*A.*) *tanabei*	2,102	40.9	41.4	11.4	6.3	−0.006	0.288
	P. (*P.*) *huazhii*	2,108	40.0	40.7	12.7	6.6	−0.009	0.316
	P. (*P.*) *pseudogigas*	2,109	40.0	41.0	12.3	6.7	−0.012	0.295
	P. (*P.*) *variegata*	2,108	40.1	41.1	12.2	6.6	−0.012	0.298
	P. (*S.*) *hani*	2,104	40.7	40.7	12.1	6.6	0.000	0.294
**D-loop region**	*A. dentata*	1,912	51.9	41.9	1.5	4.7	0.107	−0.516
	A. femorata	1,672	50.3	43.7	1.8	4.2	0.070	−0.400
	A. nagatai	1,554	49.4	44.6	1.7	4.3	0.051	−0.433
	A. setosa	1,995	50.2	42.8	1.7	5.4	0.080	−0.521
	A. spinifemorata	1,319	51.8	42.1	2.8	3.3	0.103	−0.082
	A. yifengi	1,515	50.8	43.0	2.3	3.8	0.083	−0.246
	P. (*A.*) *longipenis*	1,355	48.7	43.5	3.0	4.7	0.056	−0.221
	P. (*A.*) *tanabei*	1,084	50.6	42.3	2.4	4.7	0.089	−0.324
	P. (*P.*) *huazhii*	1,182	47.5	43.3	3.2	6.0	0.046	−0.304
	P. (*P.*) *pseudogigas*	1,158	47.5	42.7	3.8	6.0	0.053	−0.224
	P. (*P.*) *variegata*	1,134	50.2	42.1	2.8	4.9	0.088	−0.273
	P. (*S.*) *hani*	1,118	47.9	44.1	2.9	5.2	0.041	−0.284

Table S6. Nonsynonymous substitutions per nonsynonymous site (Ka), synonymous substitutions per synonymous site (Ks), and Ka/Ks (ω) values (mean ± standard deviation, SD) of the 13 PCGs in the *Amiota* and *Phortica* mitogenomes.

PCG	*Amiota*			*Phortica*		
	Ka	Ks	ω	Ka	Ks	ω
ND2	0.07310 ± 0.02511	0.31484 ± 0.03813	0.22911 ± 0.05994	0.04121 ± 0.01843	0.44908 ± 0.07819	0.09497 ± 0.04429
COX1	0.02240 ± 0.00569	0.46136 ± 0.07231	0.04936 ± 0.01396	0.01354 ± 0.00581	0.47929 ± 0.07440	0.02781 ± 0.01033
COX2	0.03733 ± 0.01383	0.35690 ± 0.05723	0.10890 ± 0.05272	0.01989 ± 0.00804	0.41700 ± 0.08408	0.04769 ± 0.01928
ATP8	0.08515 ± 0.01683	0.27083 ± 0.09634	0.36450 ± 0.16606	0.04191 ± 0.01528	0.25053 ± 0.09556	0.18193 ± 0.08914
ATP6	0.04048 ± 0.01084	0.46746 ± 0.10290	0.09053 ± 0.02977	0.02583 ± 0.01104	0.43904 ± 0.04247	0.05854 ± 0.02380
COX3	0.03276 ± 0.01007	0.40396 ± 0.08413	0.08493 ± 0.03326	0.01443 ± 0.00478	0.47062 ± 0.08853	0.03112 ± 0.01149
ND3	0.05183 ± 0.00867	0.37636 ± 0.09204	0.14624 ± 0.04529	0.04426 ± 0.01964	0.50563 ± 0.08984	0.08602 ± 0.03596
ND5	0.05822 ± 0.01502	0.35825 ± 0.03984	0.16226 ± 0.03723	0.03616 ± 0.01356	0.42760 ± 0.04295	0.08462 ± 0.03043
ND4	0.06171 ± 0.01725	0.34988 ± 0.03464	0.17897 ± 0.05528	0.03612 ± 0.01563	0.43492 ± 0.05970	0.08151 ± 0.03088
ND4L	0.03065 ± 0.01447	0.24184 ± 0.06298	0.13640 ± 0.07431	0.01354 ± 0.00661	0.39400 ± 0.06108	0.03534 ± 0.01869
ND6	0.07784 ± 0.02091	0.32163 ± 0.04968	0.24282 ± 0.05819	0.05730 ± 0.02188	0.47782 ± 0.08720	0.12112 ± 0.04654
CYTB	0.03774 ± 0.00735	0.38616 ± 0.08462	0.10049 ± 0.02328	0.02564 ± 0.00831	0.56119 ± 0.08176	0.04574 ± 0.01396
ND1	0.03304 ± 0.00579	0.35991 ± 0.05560	0.09392 ± 0.02120	0.01493 ± 0.00685	0.38131 ± 0.04933	0.03908 ± 0.01728

Table S7. Partitioning schemes and data subsets evaluated for the Bayesian inference (BI) and Maximum likelihood (ML) analysis by PartitionFinder. For the data set based on the nucleotides of the 13 PCGs and two rRNAs, best-fit selected substitution models for the PCGs (by codon positions) and rRNAs (by genes) are shown; for the data set of the amino acids based on the 13 PCGs, best-fit selected substitution models for the PCGs (by genes) are shown.

Subset	13 PCGs + two rRNAs (nucleotides)		13 PCGs (amino acids)	
	**Partitioning scheme**	**Best model**	**Partitioning scheme**	**Partitioning scheme**
1	*ATP8* 1st, *ND2* 1st, *ND6* 1st, *ND3* 1st	TIM+G	*ATP8*, *ND2*	MTART+G+F
2	*ATP8* 2nd, *ND2* 2nd	TRN+G	*COX1*, *COX3*	MTMAM+I+G+F
3	*COX1* 3rd, *ND2* 3rd	TIM+G	*COX2*, *CYTB*	MTMAM+I+G+F
4	*COX1* 1st	TRN+I+G	*ATP6*, *ND1*	MTART+I+G
5	*COX1* 2nd	F81+I+G	*ND6*, *ND3*	MTMAM+G+F
6	*COX3* 1st, *ATP6* 1st, *COX2* 1st, *CYTB* 1st	GTR+G	*ND4L*, *ND5*, *ND4*	MTART+I+G+F
7	*COX2* 2nd	F81+G		
8	*COX2* 3rd, *COX3* 3rd, *CYTB* 3rd	TIM+G		
9	*ATP8* 3rd, *ND6* 3rd, *ND3* 3rd	TRN+G		
10	*ND1* 2nd, *ATP6* 2nd, *CYTB* 2nd	TVM+I+G		
11	*ATP6* 3rd	TRN+G		
12	*COX3* 2nd	TVM+G		
13	*ND3* 2nd, *ND6* 2nd	GTR+G		
14	*ND4* 1st, *ND5* 1st	GTR+I+G		
15	*ND4* 2nd, *ND5* 2nd	GTR+I+G		
16	*ND5* 3rd, *ND4* 3rd	TIM+I+G		
17	*ND4L* 1st, *ND1* 1st	TIM+G		
18	*ND4L* 2nd	TRN		
19	*ND1* 3rd, *ND4L* 3rd	K81UF+G		
20	srRNA, lrRNA	GTR+I+G		
